# Prevalence and predictors of home and automobile smoking bans and child environmental tobacco smoke exposure: a cross-sectional study of U.S.- and Mexico-born Hispanic women with young children

**DOI:** 10.1186/1471-2458-6-265

**Published:** 2006-10-27

**Authors:** Melissa Gonzales, Lorraine Halinka Malcoe, Michelle C Kegler, Judith Espinoza

**Affiliations:** 1 Division of Epidemiology and Biostatistics, Department of Internal Medicine, and New Mexico Center for Environmental Health Sciences, University of New Mexico Health Sciences Center, Albuquerque, New Mexico, USA; 2 Masters in Public Health Program, Department of Family and Community Medicine, University of New Mexico School of Medicine, Albuquerque, New Mexico, USA; 3 Department of Behavioral Sciences and Health Education, Rollins School of Public Health, Emory University, Atlanta, Georgia, USA; 4 Environmental Health Epidemiology Bureau, New Mexico Department of Health, Albuquerque, New Mexico, USA

## Abstract

**Background:**

Detrimental effects of environmental tobacco smoke (ETS) exposure on child health are well documented. Because young children's primary exposure to ETS occurs in homes and automobiles, voluntary smoking restrictions can substantially reduce exposure. We assessed the prevalence of home and automobile smoking bans among U.S.- and Mexico-born Hispanics in the southwestern United States, and examined the influence of mother's country of birth and smoking practices on voluntary smoking bans and on child ETS exposure.

**Methods:**

U.S.- and Mexico-born Hispanic mothers of children aged 2 through 12 years were systematically sampled from health clinics in Albuquerque, New Mexico. In-person interviews were conducted with 269 mothers (75.4% response rate) to obtain information on main study outcomes (complete versus no/partial home and automobile smoking bans; child room and automobile ETS exposure) and risk factors (mother's country of birth, maternal and household smoking behaviors). Data were analyzed with chi square tests and logistic regression models.

**Results:**

Three-fourths (74–77%) of U.S.-born and 90–95% of Mexico-born mothers reported complete automobile and home smoking bans. In multivariate analyses, mother's U.S nativity, mother's current smoking, and presence of other adult smokers in the home were associated with significantly increased odds of not having a complete home or automobile smoking ban. Mother's smoking was associated with child ETS exposure both indoors (odds ratio [OR] = 3.31) and in automobiles (OR = 2.97). Children of U.S.-born mothers had increased odds of exposure to ETS indoors (OR = 3.24; 95% confidence interval [CI]: 1.37–7.69), but not in automobiles. Having complete smoking bans was associated with substantially reduced odds of child ETS exposure both indoors (OR = 0.10; 95% CI: 0.04–0.27) and in automobiles (OR = 0.14; 95% CI: 0.05–0.36).

**Conclusion:**

This study of Hispanic mothers in the southwestern U.S. indicates that there are substantial differences between U.S.- and Mexico-born mothers in the prevalence of home and automobile smoking bans, and resulting child ETS exposure. Tobacco interventions to increase smoke-free environments for U.S. Hispanic children should focus on both home and automobile smoking practices, especially among U.S.-born mothers, and utilize strategies that impact smoking practices of all household members.

## Background

The detrimental effects of environmental tobacco smoke (ETS) exposure on child health have been well documented and include chronic respiratory symptoms, asthma, decreased lung function, and middle ear disease [1-3]. Infants and young children exposed to maternal smoke are at especially high risk due to the intensity and duration of the exposure [4]. In the United States, nearly 40% of children reside in homes where adults smoke and ETS exposure is even more common among children living in poverty [5-7].

Voluntary household smoking restrictions, and especially complete home smoking bans, can substantially reduce child ETS exposure [8-12]. Studies evaluating smoking restrictions have found that U.S. Hispanic households are as likely or more likely to ban smoking in the home and automobile than non-Hispanic White households, and significantly more likely to ban smoking than African American households [13,14]. Few studies have examined automobile smoking bans among racial/ethnic groups, though existing data suggest that the prevalence of car smoking bans among U.S. Hispanics (67%) is similar to Whites, but higher than among African Americans (55%) [13]. To date no studies have examined the prevalence and degree of voluntary smoking restrictions or child ETS exposure among U.S.-born and Mexico-born Hispanics.

Hispanics are heterogeneous with respect to social and cultural characteristics as well as various health outcomes including tobacco use [15,16]. Smoking prevalence is a strong predictor of smoking bans, and recent data indicate substantial heterogeneity in smoking prevalence rates among U.S. Hispanic subgroups, particularly between English- and Spanish-speaking Hispanic women [17]. These differences in smoking rates may also extend to differences in voluntary smoking restrictions among U.S. Hispanic subgroups.

This paper examines the prevalence of home and automobile smoking bans, as well as associations of mother's country of birth and smoking practices with home and automobile smoking bans and child ETS exposure, among U.S.- and Mexico-born Hispanics residing in the southwestern United States. This research provides new information to public health professionals for increasing the adoption of home and automobile smoking bans, and for reducing ETS exposure among Hispanic children.

## Methods

Study data were collected as part of a survey of stressors, social supports, and smoking behaviors among Hispanic mothers of young children. The University of New Mexico Health Sciences Center's Human Research Review Committee approved the study protocol. During November 2003 through April 2004, study staff recruited survey participants from waiting rooms of a pediatric emergency room/urgent care clinic, a family practice and pediatric health care facility, and a Special Supplemental Nutrition Program for Women, Infants, and Children (WIC) clinic, all located in southeast Albuquerque, New Mexico.

Clinic appointment logs and sign-in sheets were used to systematically screen women who presented for clinic services. Eligible study participants included U.S.-born or Mexico-born Hispanic mothers of children aged 2 through 12 years. During screening, women were asked, "Which of the following best describes your ethnicity?". Response options were Mexican, Mexican American, Spanish New Mexican, Other Hispanic, and non-Hispanic. Women who responded 'Other Hispanic' were eligible if they were born in the United States or Mexico. A total of 357 eligible Hispanic mothers were identified. Of these, 81 declined to participate and an additional seven interviews could not be completed for other reasons, resulting in a final sample size of 269 (144 U.S.-born; 125 Mexico-born) mothers. The overall response rate was 75.4% (70% for Spanish speakers and 81% for English speakers).

### Data collection

The survey was independently translated into Spanish and verified by back translation into English by two certified translators. After obtaining written informed consent, trained bilingual interviewers conducted interviews with mothers in Spanish (n = 130) or English (n = 139). The survey included questions on maternal and household smoking behaviors and restrictions, sociodemographics, and child ETS exposure.

### Measures

To increase comparability across studies, smoking restriction and smoking behavior measures were based on items used by Kegler and Malcoe, and were similar to those used by Norman et al. and the 1999 National Health Interview Survey [12,13,18]. Home smoking restrictions were measured with one item, "Would you say family members and visitors can: (a) smoke wherever they want in your home, (b) smoke in certain rooms only, or (c) not smoke anywhere inside your home?." Automobile smoking restrictions were similarly assessed with one item, "Would you say: (a) there are no rules about smoking in your family cars, (b) smoking is sometimes allowed in a family car, or (c) smoking is never allowed in any family car?." Respondents could also indicate that they did not have a family car (n = 7).

Two questions assessed mother's smoking status. Respondents were first asked, "Have you smoked at least 100 cigarettes in your entire life?." Those who responded 'no' were classified as nonsmokers; those who responded 'yes' were asked, "How many days per week do you smoke cigarettes now?." Those who reported smoking 1–7 days per week were classified as smokers. Among smokers, the number of cigarettes smoked per day was assessed with one item, "On days you smoke, how many cigarettes do you usually smoke?." This information was combined with the number of days smoked per week to determine the number of cigarettes smoked per week. Respondents were also asked whether any other adults currently living in the home smoke cigarettes. The proportion of each respondent's friends who smoked was assessed by asking, "How many of your friends are smokers, is it: most, about half, less than half, a few, none?."

Child ETS exposure was assessed for one eligible child per household. Each respondent was asked to list the ages of her biological children who currently lived with her. One child aged 2–12 years was randomly selected (based on the child with the most recent birthday) as the 'target child' for questions regarding ETS exposure. Mothers were then asked how many days, during the past seven days, their target child had been in a room with someone who was smoking, and in a car with someone who was smoking.

Social and demographic information collected included the mother's marital status, age, education level, employment status, ethnicity, country of birth (Mexico or United States), and language preference. Data collected on monthly household income and the number of adults and children supported by that income were used to calculate the percent of the 2004 U.S. federal poverty thresholds [19], which the U.S. government uses to estimate the number of persons in poverty each year.

### Statistical analysis

The survey was designed in Teleforms v.8 (Verity Inc., Sunnyvale, CA) and optically scanned into an electronic database. Data were validated to minimize errors and transferred into SAS v8.2 (Cary, NC) for analysis. Chi square tests were used to assess univariate associations. Multiple logistic regression models were developed using non-automated stepwise modelling techniques to identify multivariate associations between study factors and the primary outcome variables: home or automobile smoking bans (complete ban versus no/partial ban), and child room or automobile ETS exposure. Variables with *p *values < 0.05 were considered statistically significant; however, all variables that were at least minimally (*p *≤ 0.25) associated with outcome variables in univariate analyses were tested for inclusion in final models.

## Results

### Participant characteristics

The majority (94%) of Mexico-born mothers preferred to be interviewed in Spanish and most U.S.-born mothers preferred English (91%). Mexico-born mothers had lived in the United States a median of 6 years (range = 0.5–35 years); 15% had lived in the United States for fewer than two years. The mean age of mothers was 29.9 (SD = 6.9) years.

There were significant differences in sociodemographic characteristics of respondents by country of birth (Table 1). More than half (56%) of mothers born in Mexico were married and 18% were single or widowed, whereas 39% of U.S.-born mothers were married and 33% were single or widowed. U.S.-born mothers were younger than those born in Mexico. Compared with U.S.-born mothers, Mexico-born mothers were substantially less likely to be formally educated beyond the 6^th ^grade or to be continuously employed. Half (48%) of Mexico-born mothers compared with one fourth (23%) of U.S.-born mothers lived below the 2004 U.S. federal poverty threshold.

Smoking characteristics also varied with mother's country of birth (Table 2). One in ten Mexico-born mothers versus three in ten U.S.-born mothers were current smokers. Likewise, U.S.-born mothers had a greater proportion of friends who smoked.

### Smoking ban prevalence

Home and automobile smoking bans were dichotomized for analyses into complete ban versus no/partial ban. Homes with complete smoking bans were reported by 85% of respondents (66% among smoking households and 96% among nonsmoking households). In univariate analyses, home smoking bans were associated with mother's country of birth and marital status, but not with mother's age or socioeconomic characteristics (Table 3). Complete home bans were less frequent among U.S.-born (77%) than Mexico-born respondents (95%). Home smoking bans were also less common among single or widowed mothers (75%) compared with those who were married (92%), living with a partner (83%), or divorced/separated (90%).

Complete bans were also associated with several smoking-related variables (Table 4). Nearly all (91%) nonsmoking mothers reported complete home smoking bans versus 63% of smoking mothers. The frequency of home smoking bans generally decreased as the proportion of mother's friends who smoked increased. Households with adult smokers other than the mother banned smoking less often (62%) than households with no other smokers (94%).

A total of 81% of mothers reported that smoking was never allowed in any family automobile. As with home smoking bans, complete automobile smoking bans were associated with mother's country of birth and other smoking-related variables, but not with socioeconomic measures (Tables 3 and 4). Complete automobile smoking bans were most common among Mexico-born and nonsmoking mothers.

### Child ETS exposure

Children were exposed to room ETS a median of 4 days in the past seven days when they lived in homes with no smoking restrictions, versus 2 days when their home had partial restrictions, and 0 days when smoking was completely banned in the home. Automobile ETS exposure was also more frequent when there were no smoking restrictions for the family automobile (median = 3 days) compared with partial or complete automobile smoking bans (median = 0 days). Children living in households with complete home or automobile smoking bans were exposed to ETS significantly fewer days than those living in households with partial or no bans (*p *< 0.001, Wilcoxon sum-rank test).

### Multivariate analyses

#### Home and automobile smoking bans

The final multivariate models predicting complete smoking bans in the home and family automobile are shown in Table 5. Compared with Mexico-born mothers, U.S.-born mothers had 3 to 6-fold lower odds of having complete smoking bans in their family automobiles (OR = 0.38; 95% CI = 0.17 – 0.89) or home (OR = 0.17, 95% CI = 0.06 – 0.50). Similarly, the odds of having automobile or home smoking bans were substantially increased among nonsmoking mothers (OR_automobile _= 5.47; 95% CI = 2.50 – 11.95; OR_home _= 2.87; 95% CI = 1.20 – 6.90) and in homes with no other adult smokers (OR_automobile _= 8.20; 95% CI = 3.75 – 17.94; OR_home _= 11.17; 95% CI = 4.56 – 27.38). Compared with married mothers, single or widowed mothers had lower odds of complete home bans (OR = 0.33; 95% CI = 0.12 – 0.93). However, mother's marital status was not independently associated with automobile smoking restrictions.

#### Child room and automobile ETS exposure

Results of the multivariate models predicting child's recent ETS exposure in a room or family automobile (Table 6) show that child room ETS exposure was positively associated with mother's country of birth (OR_U.S.-born _= 3.24; 95% CI = 1.37 – 7.69) and mother's current smoking (OR = 3.31; 95% CI = 1.47 – 7.46), and negatively associated with complete home smoking bans (OR = 0.10; 95% CI = 0.04 – 0.27). Presence of other adult smokers in the home did not remain significantly associated with child room ETS exposure in the multivariate model.

In contrast with room ETS exposure, child automobile ETS exposure was not independently associated with mother's country of birth (OR_U.S.-born _= 1.71; 95% CI: 0.64–4.56) in the final model. However, child automobile ETS exposure was similarly associated with mother's smoking (OR = 2.97; 95% CI = 1.16 – 7.62) and was substantially mitigated by complete automobile smoking bans (OR = 0.14; 95% CI = 0.05 – 0.36).

## Discussion

This study is the first to examine the prevalence and correlates of home and automobile smoking restrictions, and of child ETS exposure, among U.S.-born and Mexico-born Hispanics in the United States. We found that the adoption of complete home and automobile smoking bans were strongly associated with mother's country of birth after controlling for smoking behaviors and sociodemographic characteristics. In contrast with Mexico-born mothers, U.S.-born mothers in our sample had 6-fold greater odds of lacking a complete home smoking ban and 3-fold greater odds of lacking a complete automobile smoking ban. Given that complete smoking bans provide substantially greater protection from ETS exposure than partial bans, tobacco education programs designed to reduce child ETS exposure should consider maternal nativity an important parameter differentiating smoking restriction practices among U.S. Hispanics [20].

Young children's ETS exposure occurs primarily in homes and automobiles [21]. The overall prevalence (86%) of home smoking bans observed in our sample of U.S.- and Mexico-born Hispanic mothers in Albuquerque, New Mexico is somewhat higher than findings from studies of California Hispanics (79.9%) [13]. Only a few studies have reported on automobile smoking bans. Our observed prevalence (81%) of complete smoking bans for the family automobile was also higher than rates reported by Norman et al. among Hispanics (67%) and African Americans (55%) in California, and more than double the 38% reported by Kegler and Malcoe among rural low-income families of Native American and White young children in Oklahoma [13,14]. The higher rate of smoking bans observed in our study may reflect the presence of children in all study homes, as well as the lower smoking prevalence among U.S. Hispanic women in general and Mexico-born women in particular [11,14,22-25]. However, despite the higher prevalence of home and automobile smoking bans observed in our study, substantial opportunity for improvement remains, especially among smoking and U.S.-born Hispanic mothers.

Previous studies have demonstrated that smokers are less likely to ban home and automobile smoking [11-13,23]. Our study confirms these findings in a clinic-based sample of Hispanic families with young children. We found that complete bans were present in 63% of smoking households, in contrast with 96% of nonsmoking households. Further, our multivariate analyses showed that U.S.-born mothers and homes with adult smoker(s) other than the mother had much greater odds of *lacking *complete home and automobile smoking bans. These latter findings suggest that interventions to increase smoke-free home environments for Hispanic children in the southwestern United States should focus on U.S.-born mothers in particular and utilize strategies that impact smoking practices of all household members [26].

An important tobacco control objective of *Healthy People 2010 *is to reduce the proportion of U.S. children who are regularly exposed to tobacco smoke in the home [27]. We found that 36% of Hispanic children in our sample lived with a smoker. Our analyses further showed that complete smoking bans were associated with seven to 10-fold lower odds of room or automobile ETS exposure among Hispanic children. Several randomized controlled trials have demonstrated the effectiveness of parent counselling and coaching for decreasing children's reported ETS exposure, but only one has shown significant decreases in objective ETS exposure measures greater than those observed among controls [26,28-30]. Notably, Hovell and colleagues showed that coaching is effective in reducing ETS exposure among Latino asthmatic children where the majority of mothers were nonsmokers and of Mexican descent [26]. However, no trials have investigated the effectiveness of interventions for reducing home ETS exposure among children of U.S.-born Hispanics. Our multivariate findings, which showed that Hispanic children of U.S.-born mothers (compared with Mexico-born mothers) had three-fold increased odds of room ETS exposure, indicate that future trials should design and test ETS interventions targeting U.S.-born Hispanics with young children.

Our study is not without limitations. First, smoking restrictions and child ETS exposure were self-reported by the mother. Studies including biomarker and environmental measures of ETS have found that objective ETS exposure measures are somewhat higher than those based on self-reports [31,32]. However, we have no reason to believe that U.S.- compared with Mexico-born mothers would be more or less likely to incorrectly report their child's ETS exposure. Second, our study population was recruited from pediatric emergency/urgent care and primary care clinics that provide services to underserved populations, and not through a population-based sampling approach. To avoid selection bias, women presenting for clinic services were systematically screened for eligibility through use of appointment logs and sign-in sheets. Thus, our sample was generally representative of low-income, urban Hispanic women in the study area who utilize clinic services. Finally, we did not ask mothers if their child was exposed to ETS in their *own *home or family car, or whether they were exposed to ETS in other locations not under the control of parental smoking bans. Future studies may wish to include these questions to more fully evaluate the impact of personal smoking restriction policies on child ETS exposure.

## Conclusion

The reduction of child ETS exposure is a national priority in the United States [27]. This paper presents new findings of substantial differences between U.S.- and Mexico-born Hispanics in the prevalence of home and automobile smoking bans, and resulting child ETS exposure. As the Hispanic population of the United States grows, more women will belong to the high-risk U.S.-born group, and thus active smoking and ETS exposure will have a greater impact on the health of these women and their children. This trend could be especially significant among Mexican American women because they represent the largest and fastest growing segment of the Hispanic population in the United States [33]. The involvement of health professionals in encouraging Hispanic parents to reduce ETS exposure in the home and automobile, and ideally, to opt for smoke-free environments, is an important public health approach to reducing ETS-related morbidity. Tobacco intervention messages targeted at reducing ETS exposure among Hispanic children should consider maternal nativity an important factor influencing home and automobile smoking restriction practices.

## Competing interests

The author(s) declare that they have no competing interests.

## Authors' contributions

MG and LHM conceived of and designed the study, oversaw study implementation, analysed and interpreted the data; and drafted and revised the manuscript. MCK contributed to development of study measures and critically revised the manuscript. JE was the data manager for the study, contributed to data analysis, and revised the manuscript. All authors read and approved the final manuscript.

## Pre-publication history

The pre-publication history for this paper can be accessed here:



## Figures and Tables

**Table 1:** Sociodemographic characteristics of 269 Hispanic mothers*

**Characteristic**	**Total No. (%)^**†**^**	**Mexico-born^**‡ **^No. (%)^**†**^**	**U.S.-born No. (%)^**†**^**	***P***
Marital status				<.001
Married	126 (47)	70 (56)	56 (39)	
Live-in partner	53 (20)	29 (23)	24 (17)	
Single/Widowed	69 (26)	22 (18)	47 (33)	
Divorced/Separated	21 (8)	4 (3)	17 (12)	
Age, years				.014
<25	69 (26)	21 (17)	48 (33)	
25–28	68 (25)	32 (26)	36 (25)	
29–34	65 (24)	36 (29)	29 (20)	
≥35	67 (25)	36 (29)	31 (22)	
Education				<.001
≤6^th ^grade	36 (13)	36 (29)	0 (0)	
>6^th ^grade, <High school graduate	75 (28)	43 (34)	32 (22)	
High school graduate/GED	95 (35)	30 (24)	65 (45)	
Some college or vocational training	54 (20)	14 (11)	40 (28)	
College graduate	9 (3)	2 (2)	7 (5)	
Employment				.001
Employed consistently, full or part-time	156 (58)	58 (46)	98 (68)	
Employed intermittently	32 (12)	17 (14)	15 (10)	
Unemployed	81 (30)	50 (40)	31 (22)	
Annual household income, U.S.$				.010
<12,000	44 (16)	29 (23)	15 (10)	
12,000–18,999	65 (24)	35 (28)	30 (21)	
19,000–24,999	54 (20)	22 (18)	32 (22)	
25,000–34,999	58 (22)	23 (18)	35 (24)	
≥35,000	48 (18)	16 (13)	32 (22)	
Percent of U.S. federal poverty threshold				<.001
<100%	86 (34)	55 (48)	31 (23)	
100–185%	80 (32)	39 (34)	41 (30)	
>185%	84 (34)	21 (18)	63 (47)	

GED = General equivalency diploma.

*Some strata do not sum to 269 because of missing data.

^†^Column percent.

^‡^Includes one woman who self-identified as Mexican, but was born in Costa Rica.

**Table 2:** Smoking characteristics of 269 Hispanic mothers*

**Characteristic**	**Total No. (%)^**†**^**	**Mexico-born^**‡ **^No. (%)^**†**^**	**U.S.-born No. (%)^**†**^**	***P***
Current smoking status				<.001
Nonsmoker	216 (80)	113 (90)	103 (72)	
Smoker	53 (20)	12 (10)	41 (28)	
Cigarettes smoked per week^§^				.726
1–10	15 (28)	4 (33)	11 (27)	
11–25	15 (28)	4 (33)	11 (27)	
>25	23 (43)	4 (33)	19 (46)	
Proportion of friends who smoke				.005
None	56 (21)	33 (27)	23 (16)	
A few	109 (41)	58 (47)	51 (35)	
Less than half	37 (14)	10 (8)	27 (19)	
About half	33 (12)	12 (10)	21 (15)	
Most	33 (12)	11 (9)	22 (15)	
Other adult smokers in the home				
No	201 (75)	92 (74)	109 (76)	.693
Yes	68 (25)	33 (26)	35 (24)	

*Some strata do not sum to 269 because of missing data.

^†^Column percent.

^‡^Includes one woman who self-identified as Mexican, but was born in Costa Rica.

^§^Among mothers classified as current smokers.

**Table 3:** Home and automobile smoking bans by mother's sociodemographic characteristics*

**Characteristic**	**Home Smoking Bans**			**Automobile Smoking Bans^**‡**^**		
	
	**Complete Ban No. (%)^**†**^**	**No/Partial Ban No. (%)^**†**^**	***P***	**Complete Ban No. (%)^**†**^**	**No/Partial Ban No. (%)^**†**^**	***P***
Country of birth			<.001			.001
Mexico	118 (95)	6 (5)		107 (90)	12 (10)	
United States	111 (78)	32 (22)		105 (74)	37 (26)	
Marital status			.013			.099
Married	115 (92)	10 (8)		109 (87)	16 (13)	
Live-in partner	44 (83)	9 (17)		37 (74)	13 (26)	
Single/Widowed	52 (75)	17 (25)		51 (78)	14 (22)	
Divorced/Separated	18 (90)	2 (10)		15 (71)	6 (29)	
Age, years			.306			.265
<25	59 (86)	10 (14)		53 (80)	13 (20)	
25–28	56 (84)	11 (16)		52 (76)	16 (24)	
29–34	53 (82)	12 (18)		49 (79)	13 (21)	
≥35	61 (92)	5 (8)		58 (89)	7 (11)	
Education			.112			.157
<High school graduate	98 (88)	13 (12)		80 (77)	24 (23)	
High school graduate/GED	75 (80)	19 (20)		76 (81)	18 (19)	
College/Vocational training	56 (90)	6 (10)		56 (89)	7 (11)	
Employment			.572			.089
Employed consistently, full or part-time	130 (84)	25 (16)		129 (84)	25 (16)	
Employed intermittently	28 (88)	4 (12)		20 (67)	10 (33)	
Unemployed	71 (89)	9 (11)		63 (82)	14 (18)	
Percent of U.S. federal poverty threshold			.530			.103
<100%	73 (85)	13 (15)		60 (74)	21 (26)	
100–185%	71 (90)	8 (10)		68 (87)	10 (13)	
>185%	70 (84)	13 (16)		69 (82)	15 (18)	

GED = General equivalency diploma.

*Some strata do not sum to 269 because of missing data.

^†^Row percent.

^‡^Seven respondents were excluded who reported no family automobile; one excluded because of missing data.

**Table 4:** Home and automobile smoking bans by mother and household smoking-related characteristics*

**Characteristic**	**Home Smoking Bans**			**Automobile Smoking Bans^**‡**^**		
	
	**Complete Ban No. (%)^**†**^**	**No/Partial Ban No. (%)^**†**^**	***P***	**Complete Ban No. (%)^**†**^**	**No/Partial Ban No. (%)^**†**^**	***P***
Current smoking status			<.001			<.001
Nonsmoker	196 (91)	19 (9)		190 (90)	20 (10)	
Smoker	33 (63)	19 (37)		22 (43)	29 (57)	
Cigarettes smoked per week^§^			.573			.285
1–10	11 (73)	4 (27)		9 (60)	6 (40)	
11–25	9 (64)	5 (36)		5 (38)	8 (62)	
>25	13 (57)	10 (43)		8 (35)	15 (65)	
Proportion of friends who smoke			.002			<.001
None	53 (95)	3 (5)		50 (94)	3 (6)	
A few	99 (92)	9 (8)		95 (89)	12 (11)	
Less than half	27 (75)	9 (25)		29 (78)	8 (22)	
About half	25 (76)	8 (24)		22 (69)	10 (31)	
Most	24 (73)	9 (27)		15 (48)	16 (52)	
Other adult smoker(s) in home			<.001			<.001
No	187 (94)	12 (6)		174 (89)	21 (11)	
Yes	42 (62)	26 (38)		38 (57)	28 (42)	

*Some strata do not sum to 269 because of missing data.

^†^Row percent.

^‡ ^Seven respondents were excluded who reported no family automobile; one excluded because of missing data.

^§^Among mothers classified as current smokers.

**Table 5:** Final logistic regression models predicting complete smoking bans in the home and family automobile

**Predictor**	**Complete Home Smoking Ban**	**Complete Automobile Smoking Ban**
	
	**Adjusted OR (95% CI)**	**Adjusted OR (95% CI)**
Mother's country of birth		
Mexico	1.00	1.00
United States	0.17 (0.06 – 0.50)	0.38 (0.17 – 0.89)
Mother's current smoking status		
Smoker	1.00	1.00
Nonsmoker	2.87 (1.20 – 6.90)	5.47 (2.50 – 11.95)
Other adult smoker(s) in home		
Yes	1.00	1.00
No	11.17 (4.56 – 27.38)	8.20 (3.75 – 17.94)
Marital status		
Married	1.00	... *
Live-in partner	0.62 (0.20 – 1.96)	
Single/Widowed	0.33 (0.12 – 0.93)	
Divorced/Separated	1.00 (0.16 – 6.36)	

OR = odds ratio; CI = confidence interval.

*Variable not included in final model because it did not retain statistical significance.

**Table 6:** Final logistic regression models predicting target child's exposure to environmental tobacco smoke (ETS) in a room or automobile

**Predictor**	**Child Room ETS Exposure**	**Child Automobile ETS Exposure**
	
	**Adjusted OR (95% CI)**	**Adjusted OR (95% CI)**
Mother's country of birth		
Mexico	1.00	1.00
United States	3.24 (1.37 – 7.69)	1.71 (0.64 – 4.56)
Mother's current smoking status		
Nonsmoker	1.00	1.00
Smoker	3.31 (1.47 – 7.46)	2.97 (1.16 – 7.62)
Other adult smoker(s) in home		
No	1.00	1.00
Yes	2.18 (0.92 – 5.14)	1.92 (0.77 – 4.75)
Complete home smoking ban		
No	1.00	... *
Yes	0.10 (0.04 – 0.27)	
Complete automobile smoking ban		
No	... *	1.00
Yes		0.14 (0.05 – 0.36)

OR = odds ratio; CI = confidence interval.

*Variable not included in final model because it did not retain statistical significance.

## References

[B1] Jaakkola JJ, Jaakkola MS (2002). Effects of environmental tobacco smoke on the respiratory health of children. Scand J Work Environ Health.

[B2] Cook DG, Strachan DP (1999). Summary of effects of parental smoking on the respiratory health of children and implications for research. Thorax.

[B3] Mannino DM, Homa DM, Redd SC (2002). Involuntary smoking and asthma severity in children: data from the Third National Health and Nutrition Examination Survey. Chest.

[B4] Tager IB, Ngo L, Hanrahn JP (1995). Maternal smoking during pregnancy: Effects on lung function during the first 18 months of life. Am J Respir Crit Care Med.

[B5] Gergen PJ, Fowler JA, Maurer KR, Davis WW, Overpeck MD (1998). The burden of environmental tobacco smoke exposure on the respiratory health of children 2 months through 5 years of age in the United States: Third National Health and Nutrition Examination Survey, 1988 to 1994. Pediatrics.

[B6] Mannino DM, Caraballo R, Benowitz N, Repace J (2001). Predictors of cotinine levels in U.S. children: Data from the Third National Health and Nutrition Examination Survey. Chest.

[B7] Schuster M, Franke T, Pham C (2002). Smoking patterns of household members and visitors in homes with children in the United States. Arch Pediatr Adolesc Med.

[B8] Biener L, Cullen D, Xiao Z, Hammond SK (1997). Household smoking restrictions and adolescents' exposure to environmental tobacco smoke. Prev Med.

[B9] Wakefield M, Banham D, Martin J, Ruffin R, McCaul K, Badcock N (2000). Restrictions on smoking at home and urinary cotinine levels among children with asthma. Am J Prev Med.

[B10] Berman BA, Wong GC, Bastani R, Hoang T, Jones C, Goldstein DR, Bernert JB, Hammond SK, Tashkin D, Lewis MA (2003). Household smoking behavior and ETS exposure among children with asthma in low-income, minority households. Addict Behav.

[B11] Pizacani BA, Martin DP, Stark MJ, Koepsell TD, Thompson B, Diehr P (2003). Household smoking bans: which households have them and do they work?. Prev Med.

[B12] Kegler MC, Malcoe LH (2002). Smoking restrictions in the home and car among rural Native American and White families with young children. Prev Med.

[B13] Norman GJ, Ribisl KM, Howard-Pitney B, Howard KA (1999). Smoking bans in the home and car: Do those who really need them have them?. Prev Med.

[B14] Gilpin EA, White MM, Farkas AJ, Pierce JP (1999). Home smoking restrictions: which smokers have them and how are they associated with smoking behavior. Nicotine Tob Res.

[B15] Borrell LN (2005). Racial identity among Hispanics: Implications for health and well-being. Am J Public Health.

[B16] Caraballo RS, Lee CW (2004). Tobacco use among Mexicans and their descendants in the United States. Salud Publica Mex.

[B17] Maher JE, Boysun MJ, Rohde K, Stark MJ, Pizacani BA, Dilley J, Mosbeak CH, Pickle KE (2005). Are Latinos really less likely to be smokers? Lessons from Oregon. Nicotine Tob Res.

[B18] Centers for Disease Control and Prevention (2001). Cigarette smoking among adults – United States, 1999. Morbid Mortal Wkly Rep.

[B19] U.S. Census Bureau Poverty Thresholds 2004. http://www.census.gov/hhes/poverty/threshld/thresh04.html.

[B20] Blackburn C, Spencer N, Bonas S, Coe C, Dolan A, Moy R (2003). Effect of strategies to reduce exposure of infants to environmental tobacco smoke in the home: cross sectional survey. BMJ.

[B21] Klerman LV (2004). Protecting children: reducing their environmental tobacco smoke exposure. Nicotine Tob Res.

[B22] King G, Mallett R, Kozlowski L, Bendel RB, Nahata S (2005). Personal space smoking restrictions among African Americans. Am J Prev Med.

[B23] Okah FA, Choi WS, Okuyemi KS, Ahluwalia JS (2002). Effect of children on home smoking restriction by inner-city smokers. Pediatrics.

[B24] Centers for Disease Control and Prevention (2005). Cigarette smoking among adults – United States, 2003. Morbid Mortal Wkly Rep.

[B25] Wilkinson AV, Spitz MR, Strom SS, Prokhorov AV, Barcenas CH, Cao Y, Saunders KC, Bondy ML (2005). Effects of nativity, age at migration, and acculturation on smoking among adult Houston residents of Mexican descent. Am J Public Health.

[B26] Hovell MF, Meltzer SB, Wahlgren DR, Matt GE, Hofstetter CR, Jones JA, Meltzer EO, Bernert JK, Pirkle JL (2002). Asthma management and environmental tobacco smoke exposure reduction in Latino children: a controlled trial. Pediatrics.

[B27] Healthy People Volume II (2^nd ^edition). Objectives for Improving Health, Part B, Focus Area 27: Tobacco Use. http://www.healthypeople.gov/Document/HTML/Volume2/27Tobacco.htm#_Toc489766224.

[B28] Hovell MF, Zakarian JM, Matt GE, Hofstetter CR, Bernert JT, Pirkle J (2000). Effect of counseling mothers on their children's exposure to environmental tobacco smoke: randomised controlled trial. BMJ.

[B29] Emmons KM, Hammond SK, Fava JL, Velicer WF, Evans JL, Monroe AD (2001). A randomized trial to reduce passive smoke exposure in low-income households with young children. Pediatrics.

[B30] Wilson SR, Yamada EG, Sudhakar R, Roberto L, Mannino D, Mejia C, Huss N (2001). A controlled trial of an environmental tobacco smoke reduction intervention in low-income children with asthma. Chest.

[B31] Emmons KM, Wong M, Hammond SK, Fava JL, Velicer WF, Monroe AD, Evans JL (2001). Intervention and policy issues related to children's exposure to environmental tobacco smoke. Prev Med.

[B32] Sexton K, Adgate JL, Church TR, Hecht SS, Ramachandran G, Greaves IA, Fredrickson AL, Ryan AD, Carmella SG, Geisser MS (2004). Children's exposure to environmental tobacco smoke: using diverse exposure metrics to document ethnic/racial differences. Environ Health Perspect.

[B33] Ramirez R, De la Cruz GP The Hispanic Population in the United States: March 2002. Washington, DC: U.S. Census Bureau. Current Population Reports, P20-545. http://www.census.gov/prod/2003pubs/p20-545.pdf.

